# Wedelolactone inhibits LPS-induced pro-inflammation via NF-kappaB Pathway in RAW 264.7 cells

**DOI:** 10.1186/1423-0127-20-84

**Published:** 2013-10-31

**Authors:** Fang Yuan, Jie Chen, Ping-ping Sun, Su Guan, Jing Xu

**Affiliations:** 1 Department of Pharmacology, School of Pharmacy, Guangdong Pharmaceutical University, Guangzhou 510006, China; 2 Department of Pharmacy, the First Affiliated Hospital of Sun Yat-sen University, Guangzhou 510080, China; 3 School of Bioscience and Biotechnology, South China University of Technology, Guangzhou 510006, China; 4 Department of Pharmacy, the Third Affiliated Hospital of Southern Medical University, Guangzhou 510630, China

**Keywords:** Wedelolactone (WEL), Popolysaccharide (LPS), Inflammation, Nuclear factor-κB (NF-κB), Macrophage

## Abstract

**Background:**

Wedelolactone (WEL), a major coumestan ingredient in *Wedelia chinensis*, has been used to treat septic shock, hepatitis and venom poisoning in traditional Chinese medicines. The objective of the study was to elucidate the anti-inflammatory effects and mechanism of WEL with a cellular model of lipopolysaccharide (LPS)-induced RAW 264.7 cells.

**Results:**

To study the role of WEL in pro-inflammation, we measured key inflammation mediators and end products including nitric oxide (NO), prostaglandin E2 (PGE2), inducible nitric oxide synthase (iNOS), cyclooxygenase-2 (COX-2) and tumor necrosis factor-α (TNF-α) by using the Griess method, enzyme linked immunosorbent assay (ELISA) and Western blotting. Nuclear factor-kappaB (NF-κB) transcription activity was detected by luciferase reporter assay. The important pro-inflammatory transcription factors, NF-κB p65 and inhibitory kappaB alpha (IκB-α); and mitogen-activated protein kinases (MAPKs), including extracellular signal-regulated kinase (ERK), c-Jun N-terminal kinase (JNK) and p38 MAPK (p38) were analyzed by Western blotting. Our study showed that WEL (0.1, 1, 10 μM) significantly inhibited the protein expression levels of iNOS and COX-2 in LPS-stimulated cells, as well as the downstream products, including NO, PGE2 and TNF-α. Moreover, WEL also inhibited LPS-induced NF-κB p65 activation via the degradation and phosphorylation of IκB-α and subsequent translocation of the NF-κB p65 subunit to the nucleus.

**Conclusions:**

Our results revealed that WEL has a potential to be a novel anti-inflammatory agent targeting on the NF-κB signaling pathway.

## Background

**Figure 1:**
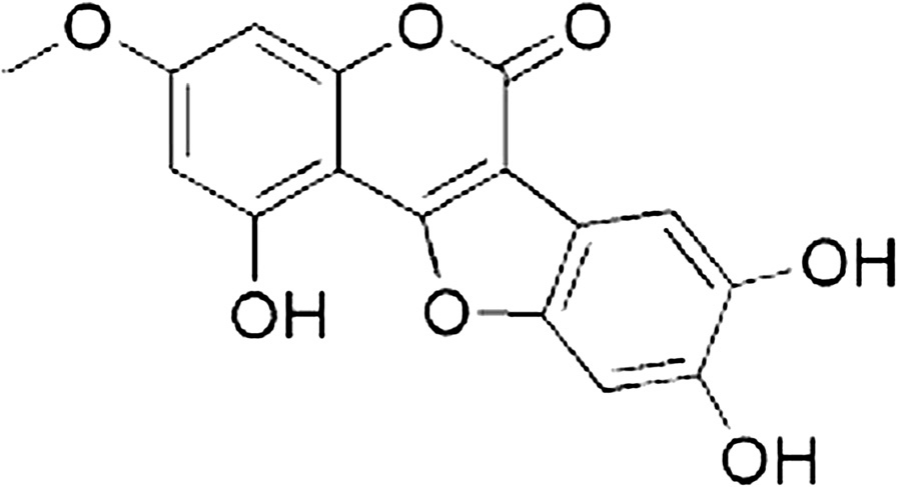
Chemical structure of WEL.

Wedelolactone (WEL), a common ingredient in *Wedelia chinensis* and *Eclipta prostrata,* belongs to the flavonoids category of phytoestrogens [1,2] (structure shown in Figure 1). As a perennial herbal, WEL has been widely used to treat septic shock, hepatitis and venom poisoning in China [3-5]. Previous studies have shown that WEL has diverse pharmacological effects such as antihepatotoxic, antiandrogenic and anti-human immunodeficiency activities [6-8]. Kobori M *et al.* demonstrated that WEL inhibits NF-kappaB (NF-κB) pathway by directly blocking phosphorylation and degradation of inhibitory kappaB alpha (IκBα) [9]. Based on this result, Ruhland A *et al.* reported that WEL can reverse host cell resistance to parasite-induced apoptosis by inhibiting NF-κB signaling pathway [10]. Other results showed that WEL inhibits adipogenesis via activation of the ERK pathway [1]. Both WEL and demethyl-wedelolactone (DWL) showed trypsin inhibition effects in vitro [11]. Taken together, WEL has been identified as an anti-NF-κB translocation, growth inhibitory and pro-apoptotic agent in differentiated and cancer cells [1,6,9,10]. However, the precise mechanisms of its anti-inflammation effects have not been completely delineated.

Inflammation is an important host response to foreign challenge or tissue injury, which leads to the restoration of tissue structure and function [12]. During the process, the activation of immune cells induced by pro-inflammatory cytokines up-regulates inflammation [13,14]. It is well known that macrophages, together with neutrophils and dendritic cells, play an important role in the innate immune response [15]. The key inflammatory mediators such as nitric oxide (NO), inducible nitric oxide synthase (iNOS), prostaglandin E_2_ (PGE_2_) and cyclooxygenase-2 (COX-2) and pro-inflammatory cytokine such as tumor-necrosis factor α (TNF-α) can be released by activated macrophages [16]. Lipopolysaccharide (LPS), a cell wall component of Gram-negative bacteria, has been reported to activate macrophages to produce inflammatory mediators such as iNOS, TNF-α and COX-2, mimicking the inflammatory reaction in *vivo*[14]. LPS triggers a serial of signal transduction events which lead to the activation of NF-κB and mitogen-activated protein kinase (MAPK) signaling pathway [17]. Herein, LPS induced macrophages is a well-established model for innate immunity study [18]. NF-κB has a pivotal role in immune and inflammatory responses through the regulation of pro-inflammatory cytokines, adhesion molecules, chemokines, growth factors and inducible enzymes, such as COX-2 and iNOS [12]. The activation of NF-κB in response to pro-inflammatory stimuli such as TNF-α has been characterized extensively. TNF-α stimulates the phosphorylation, ubiquitylation and subsequent degradation of inhibitor of IκBα [12]. Mitogen-activated protein kinases (MAPKs) play a key role in the signaling pathways of cell proliferation, differentiation, survival, apoptosis and extracellular signal transduction to the nucleus [19,20]. MAPKs can be activated by Toll-like receptor 4 (TLR4) leading to the activation of nuclear translocation of NF-κB and finally initiates pro-inflammatory responses [21]. NF-κB is activated by phosphorylation of IκBα via activation of MAPKs such as ERK1/2, JNK and p38 subfamilies, and then migrates into the nucleus and activates the expression of inflammatory cytokines and mediators [12,19,22]. The activation of NF-κB in response to pro-inflammatory stimuli such as TNF-α through phosphorylation of IκBα results in the NF-κB p65–p50 heterodimer to migrate into the nucleus and up-regulating the expression of pro-inflammatory and anti-apoptotic genes [12].

In the present study, we investigated the anti-inflammatory effects of WEL in LPS-stimulated RAW 264.7 cells and examined whether WEL could inhibit inflammatory responses via suppression of the NF-κB and MAPKs signaling pathways.

## Methods

### Cell culture and cell viability assay

Raw 264.7 cell line was obtained from cell bank, Institute of Biochemistry and Cell Biology (Shanghai, China). Raw 264.7 cells were cultured in DMEM with 10% fetal bovine serum, in an incubator at 37°C, 5% CO_2_ and 95% humidity. The cyctotoxic effects of WEL were evaluated in absence or presence of LPS by MTT assay. WEL was dissolved in 10% dimethyl sulfoxide (DMSO) and added directly to culture media before the addition of LPS. The final concentration of DMSO never exceeded 0.1%.

### Measurement of NO levels

The nitrite concentration in the culture medium was measured by a Griess reaction test. Cells were plated as a density of 2 × 10^6^ cells/well in 24-well culture plates and pretreated with or without indicated concentrations of WEL (0.1, 1, 10 μM) or N-nitro-L-arginine methyl ester (L-NAME) (100 μM) for 12 h, and then incubated with LPS (1 μg/ml). 100 μM L-NAME, an inhibitor of NO, was used as a positive control. After 20 h incubation, cells were washed three times to remove non-adherent cells. Then, 100 μl of the Griess reagent was mixed with an equal volume of cell supernatant, the optical density at 540 nm was measured and the concentration of nitrite was calculated according to the standard curve generated from known concentrations of sodium nitrite.

### Measurement of PGE2 levels

RAW 264.7 macrophages were subcultured in 24-well plates and pretreated with or without indicated concentrations of WEL (0.1, 1, 10 μM) for 12 h or DX (0.1 μM) for 1 h, then incubated with LPS (1 μg/ml) for 20 h. The accumulated PGE_2_ in the culture medium was measured using ELISA Kit (Cayman Chemical Company, Michigan, USA) according to the manufacturer’s instructions. 0.1 μM DX was used as a positive control.

### Measurement of TNF-α levels

The effects of WEL on the production of TNF-α were measured by ELISA. 2 × 10^6^ RAW 264.7 cells (1% serum starved) were seeded on 24-well plate at a density of 2 × 10^6^ per well for over-night. Cells were pre-incubated with WEL (0.1, 1, 10 μM) or DX (0.1 μM) for 1 h, then stimulated with 1 μg/ml LPS for another 20 h. The cytokine concentrations were calculated according to the standard curve using recombinant cytokines in each ELISA kits. All measurements above were performed in triplicate.

### Transient transfection and luciferase reporter assay

NF-κB reporter constructs were purchased from Clontech Laboratories, Inc. (Palo Alto, CA, USA). For the reporter assay, cells were seeded into 24-well plates at a density of 5 × 10^5^ cells per well in 500 μl of DMEM without antibiotics and incubated overnight. The cells in each well were transiently transfected with 200 ng of luciferase reporter construct and 50 ng of internal control plasmid of the pCMV-β-galactosidase reporter plasmid or empty expression vector pcDNA3 using lipofectamine TM 2000 reagent according to the manufacturer’s procedures (Invitrogen, Carlsbad, CA, USA). Six hours after transfection, the cells were washed with phosphate-buffered saline to remove LiptofectamineTM 2000 complexes and then supplied with fresh medium (supplemented with fetal calf serum and phenol red free) and treated with WEL (0.1, 1 and 10 μM) for 12 h before stimulation with LPS (1 μg/ml) for 20 h. Subsequently, luciferase activities were measured in cell lysates using Dual Luciferase Reporter reagents following manufacturer’s instruction (Promega, Madison, WI, USA).

### Western blotting analysis

After treatment with various concentrations of WEL in presence or absence of 1 μg/mL LPS, cells were analyzed by immunoblotting. The treated cells were washed and scraped into cold phosphate-buffered saline (PBS) and centrifuged at 500 × g at 4°C. The cell pellets were resuspended in lysis buffer and centrifuged to yield whole-cell lysates [23]. 20 μg protein for each sample was separated by SDS-polyacrylamide gels with electrophoresis (Bio-Rad, Hercules, CA, USA) and the gel was transferred to PVDF membrane. The membrane was blocked with 10% skim milk for 1 h and then incubated overnight at 4°C with 1:2000 dilution of the corresponding primary antibody. After washing, the membranes were incubated with the appropriate secondary antibody conjugated to horseradish peroxidase. The membrane was immersed in the enhanced chemiluminescence solution for 60 sec. The gel images were visualized using Chem-Doc (Bio-Rad, Hercules, CA) and densitometric analysis was performed with Quantity One 1-D Analysis software (Bio-Rad). The results are representative of three independent experiments.

### Drugs and solutions

WEL (Purity ≥ 95%, by HPLC) (Xidian Pharmaceutical Co., Ltd., Jilin, China) HEPES, LPS, N-nitro-L-arginine methyl ester and lipopolysaccharide (L-NAME) and 3-(4,5-dimethylthiazol-2-yl)-2,5-diphenyl-tetrazolium bromide (MTT) (Sigma, St. Louis, MO, USA). Dulbecco’s modified Eagle’s medium (DMEM) and bovine serum albumin (BSA) (Gibco BRL, Gaithersburg, MD, USA). Griess reaction kit for Nitric Oxide (NO) (Jiancheng Co., Ltd., Nanjing, China). ELISA kits for detecting TNF-α (R&D Systems, Inc., USA). PGE_2_ ELISA Kit was obtained from Cayman Chemical Company (Ann Arbor, MI, USA). Trizol reagent (Invitrogen, Carlsbad, CA, USA). Antibodies specific for COX-2, iNOS, phospho-IκBα, NF-κBp65, phospho-ERK1/2 and glyceraldehydes 3-phosphate dehydrogenase (GADPH) (Santa Cruz Biotechnology, Inc., Santa Cruz, CA, USA). Antibodies specific for MAPK family proteins (ERK1/2, phospho-p38, p38, phospho-JNK, JNK) (Cell Signaling Technology, Inc., Beverly, MA, USA). All other reagents were of analytical grade.

### Statistical analysis

The results were expressed as mean ± standard error of the mean (SEM) with the indicated number (n) of experiments. Differences between groups for continuous variables were evaluated with analysis of variance (ANOVA) and differences between two groups were analyzed using unpaired Student’s *t-*test (SPSS version 10.0; Chicago, IL). Statistical significance was set as *p* < 0.05.

## Results

### Effects of WEL on cell viability

**Figure 2:**
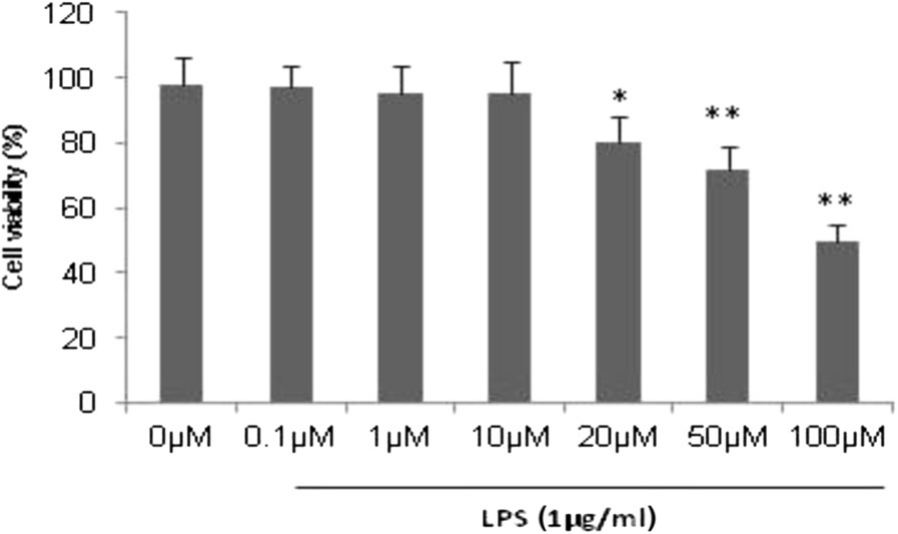
**Effects of WEL on the viability of RAW 264.7 cells.** Cell viability was measured by MTT assay. The values were presented as mean ± SEM of three independent experiments, n = 6 per experiment. ^*^p < 0*.*05 *vs*. control group, ^*^^*^p < 0.01 vs. control group.

The cytotoxicity of WEL in RAW 264.7 cells was measured by MTT assay (Figure 2). The results showed that WEL did not affect cell viability at a concentration of 0.1 μM to 100 μM regardless of the presence of LPS for 20 h. Therefore, a concentration of 0.1 to 10 μM of WEL was used in all experiments.

### Effects of WEL on NO and PGE2 production in LPS-stimulated cells

**Figure 3:**
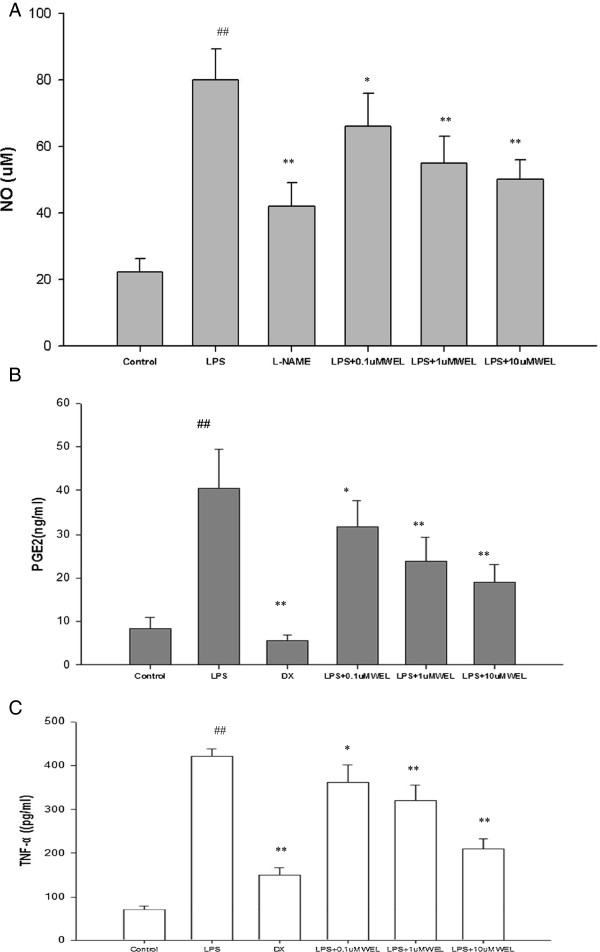
**Effects of WEL on LPS-induced NO, PGE2 and TNF-α production in RAW 264.7 macrophages. (A)** Cells were treated with the indicated concentrations of WEL (0, 0.1, 1, or 10 μ M) or 100 μM L-NAME for 12 h, respectively, and then incubated with LPS (1 μg/ml) for 20 h. 100 μM L-NAME group was set as positive control. The nitrite production was measured by the Griess reaction. **(B, C)** Cells were treated with the indicated concentrations of WEL 12 h or 0.1 DX (0.1 μM) for 1 h, respectively, and then incubated with LPS (1 μg/ml) treatment for 20 h. The PGE2 and TNF-α concentration were determined by ELISA kit. The values were presented as mean ± SEM. of three independent experiments. n = 6 per experiment. ^*^p < 0*.*05 *vs.* LPS group and ^**^p < 0.01 vs. LPS group.^*#*^*p <* 0*.*05 *vs.* none LPS control group. ^*##*^*p <* 0*.*01 *vs*. none LPS control group.

The potential anti-inflammatory effects of WEL on LPS-stimulated NO and PGE_2_ production were examined in RAW 264.7 macrophages by pretreating cells with various concentrations of WEL for 12 h before stimulation with 1 μg/mL LPS for 20 h. NO and PGE_2_ concentrations in the culture medium were measured by Griess reagent and ELISA, respectively. As shown in Figure 3, NO and PGE_2_ production was remarkably induced in LPS-stimulated RAW 264.7 macrophages when compared with un-stimulated negative controls, while pretreatment with WEL significantly prevented this increase in a dose-dependent manner. This inhibitory effect was achieved with non-cytotoxic concentrations of WEL.

### Effects of WEL on TNF-α production in LPS-stimulated cells

To study the effects of WEL on LPS-induced inflammatory related cytokine production, such as TNF-α production in RAW 264.7 cells, cells were pretreated for 12 h with various concentrations of WEL, followed by treatment with LPS (1 μg/ml) for 20 h. The production of TNF-α induced by LPS was evaluated by ELISA. Our result showed that WEL dose-dependently blocked the expression of the pro-inflammatory cytokine TNF-α (Figure 3).

### Effects of WEL on iNOS and COX-2 protein expression in LPS-stimulated cells

**Figure 4:**
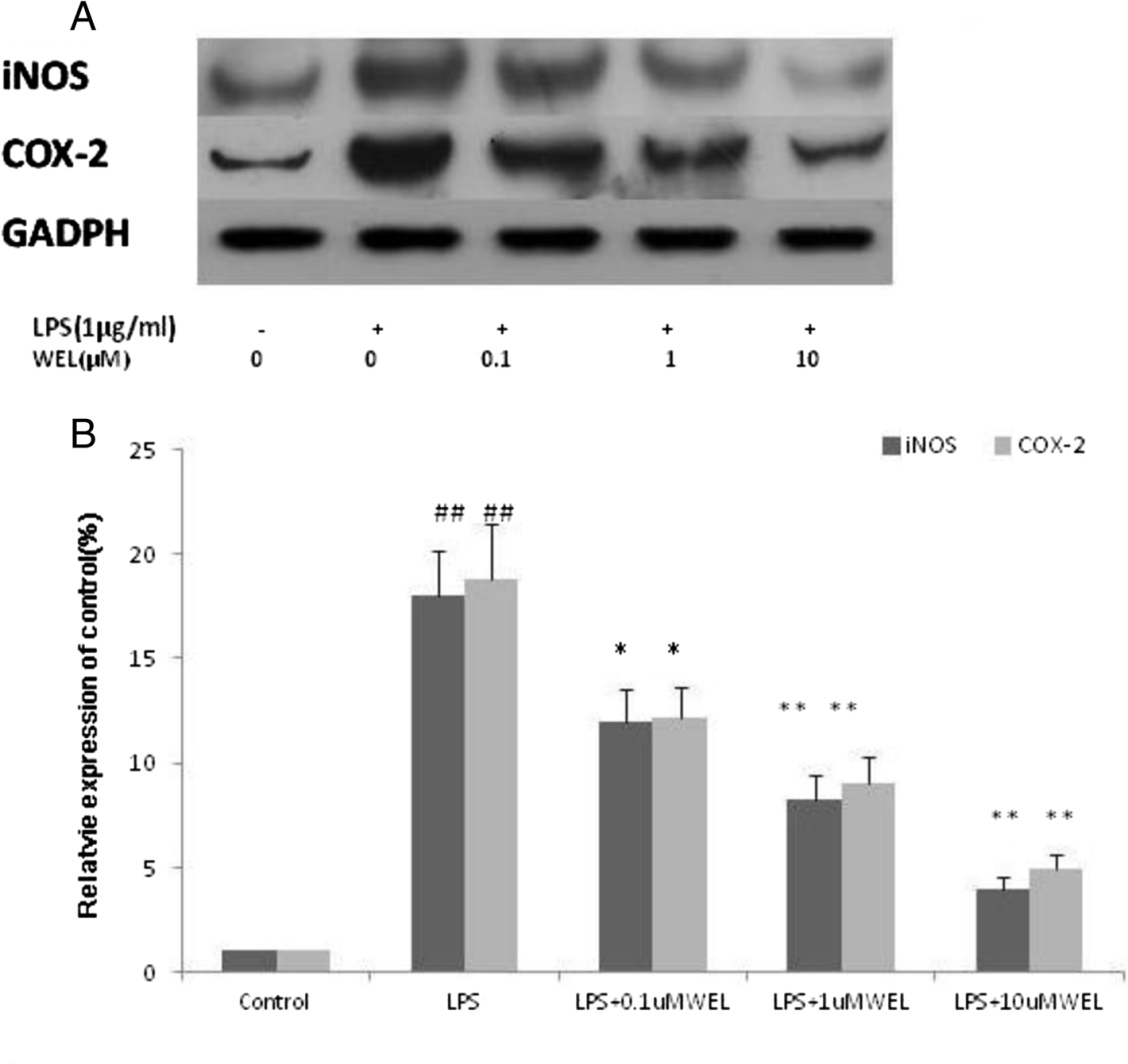
**Effects of WEL on iNOS and COX-2 protein expression in LPS-induced cells. (A)** Cells were pre-incubated with WEL (0, 0.1, 1, or 10 μ M) for 12 h, and then incubated with LPS (1 μg/ml) for 20 h. The total protein lysate was subjected to Western blotting. **(B)** The ratio of immunointensity between the iNOS, COX-2 and the GAPDH was calculated. A representative blot of each experiment is shown with the densitometric analysis corresponding to the mean ± SEM. of three independent experiments. n = 6 per experiment. ^*^p < 0.05 vs. LPS group and ^**^p < 0.01 vs. LPS group*.*^*#*^*p <* 0*.*05 *vs.* none LPS control group. ^*##*^*p <* 0*.*01 *vs.* none LPS control group.

Based on the findings above, we investigated whether the inhibition of WEL on NO and PGE_2_ production was related to down-regulation of iNOS and COX-2. Cells were pretreated with the indicated concentration of WEL for 12 h followed with LPS (1 μg/ml) treatment for another 20 h. The protein levels of iNOS and COX-2 were significantly up-regulated in response to LPS, and WEL inhibited the expression of these proteins in a dose-dependent manner (Figure 4). These results showed that WEL was able to inhibite the expression of iNOS and COX-2 enzymes, which in turn reduce the production of NO and PGE_2_, the two key mediators of inflammation, respectively.

### Effects of WEL on LPS-mediated NF-κB transcriptional activity via suppression of IκB-α degradation and nuclear translocation of the p65 and p50 subunits in RAW 264.7 cells

**Figure 5:**
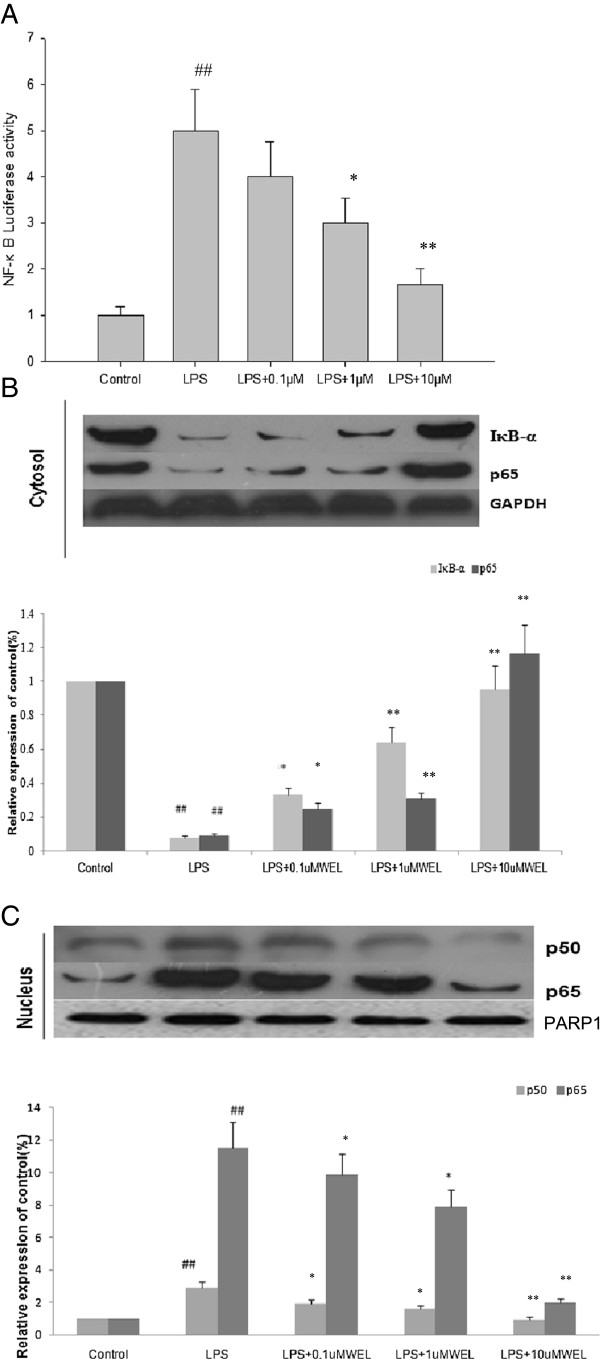
**Effects of WEL on LPS-induced NF-κB transcriptional activity via suppression of IκB-α degradation and nuclear translocation of the p65 and p50 subunits in RAW 264.7 cells. (A)** Cells were transiently co-transfected with pNF-κB-Luc reporter plasmid and then were pretreated with the indicated concentrations of WEL for 12 h. LPS (1 μg/ml) was then added and cells were further incubated for 20 h. The cells were harvested and then the luciferase activities were determined by using the dual luciferase report assay system. **(B, C)** Cells were pretreated with the indicated concentrations of WEL for12 h and then stimulated with LPS (1 μg/ml) treatment for 30 min. Cytosol **(B)** and nuclear **(C)** protein were determined by Western blot assay using anti-IκB-α, NF-κB p50, and NF-κB p65. GAPDH and PARP1 were used as internal controls for Western blotting. A representative blot of each experiment was shown with the densitometric analysis corresponding to the mean ± SEM. of three independent experiments. n = 6 per experiment. ^*^p < 0.05 vs. LPS group and ^**^p < 0.01 vs. LPS group*.*^*#*^*p <* 0*.*05 *vs.* none LPS control group. ^*##*^*p <* 0*.*01 *vs.* none LPS control group.

NF-κB plays a pivotal role in regulation of the expression of iNOS, COX-2 and inflammatory cytokines such as TNF-α [12]. The heteromeric NF-κB complex is sequestered in the cytoplasm as an inactive precursor, combined with an inhibitory IκB-α protein. Activation of NF-κB, an important transcription factor in the inflammatory response, occurs after the phosphorylation, ubiquitination and proteolytic degradation of IκB-α [24]. To investigate the underlying mechanism of the inhibition of WEL on iNOS and COX-2 protein expression in LPS-stimulated cells, luciferase reporter assay was used to explore the effects of WEL on NF-κB-dependent reporter gene expression following LPS treatment. RAW 264.7 cells were transiently cotransfected with a pNF-κB-leu reporter vector with four spaced NF-κB-binding sites into the pLuc-promoter vector and then stimulated with 1 μg/ml LPS with or without WEL. WEL significantly reduced the level of NF-κB luciferase activity induced by LPS in a dose-dependent manner (Figure 5A). To further investigate whether WEL regulates the NF-κB pathway, the cytoplasmic protein level of IκB-α was measured by western blotting after cells were pretreated with the indicated concentrations of WEL for 12 h and stimulated with LPS (1 μg/ml) for 30 min. The results showed that WEL inhibited the phosphorylation and degradation of the IκB-α protein after LPS treatment. Because p65 and p50 are the major subunits of the NF-κB heterodimer, the translocation of p65 and p50 subunits from the cytoplasm to the nucleus after being released from IκB-α were investigated. As shown in Figure 5B and C, the concentrations of p65 and p50 subunits were decreased in the cytoplasm and increased in nucleus after LPS treatment, pretreatment with WEL reversed these trends in a dose-dependent manner. Taken together, these findings demonstrated that WEL suppressed the expression of iNOS and COX-2 at least in part via NF-κB-dependent mechanism.

### Effects of WEL on the activation of ERK1/2, JNK and p38 in LPS-stimulated cells

**Figure 6:**
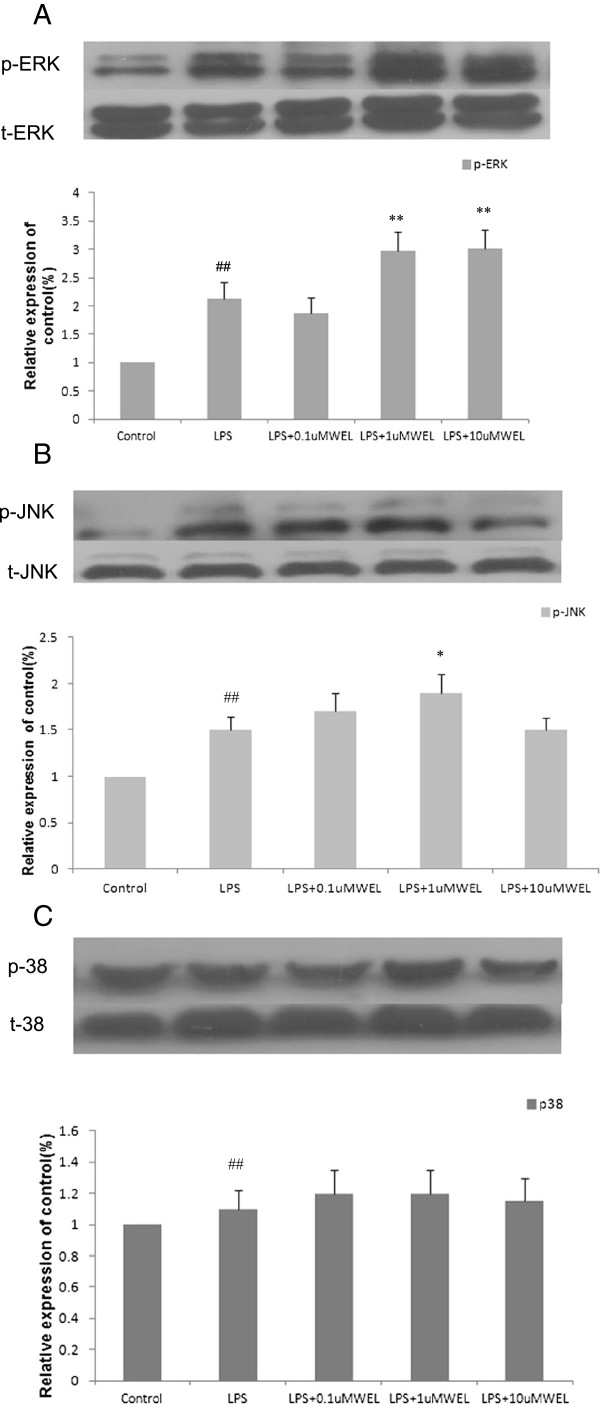
**Effects of the WEL on the activation of ERK1/2 (A), JNK (B) and p38 (C) in LPS-stimulated cells.** Cells were pre-incubated with WEL for 12 h, and then incubated with LPS (1 μg/mL) for 30 min. The total protein lysate was subjected to Western blotting analysis. The ratios of immunointensity of p-ERK1/2, p-JNK and p-p38 were calculated, respectively. Total ERK1/2, JNK and p38 (t-ERK1/2, t-JNK and t-p38) were used as a control of the protein amount in the same samples. A representative blot of each experiment is shown with the densitometric anaylsis corresponding to the mean ± SEM. of three independent experiments. n = 6 per experiment. *p < 0.05 *vs.* LPS group and **p < 0.01 *vs.* LPS group. ^*#*^*p* < 0.05 *vs.* none LPS control group. ^*##*^*p* < 0.01 *vs.* none LPS control group.

Three MAPKs, ERK, p38 and JNK, are known to be activated by LPS. MAPKs play an important role in the transcriptional regulation of LPS-induced expression of iNOS and COX-2 via activation of the transcription factor NF-κB [25]. Thus, we investigated the effect of WEL on the activation of ERK1/2, JNK and p38. After cells were pretreated with the indicated concentrations of WEL for 12 h and stimulated with LPS (1 μg/ml) for 30 min, the expression of ERK1/2, JNK, and p38 was analyzed by Western blotting. As shown in Figure 6, WEL pretreatment obviously increased phosphorylation of ERK1/2 (p-ERK1/2) and slightly enhanced phosphorylation of JNK (p-JNK). At the same time, WEL was not observed to have any effect on the LPS-induced phosphorylation of p38 MAPK. These results indicated that the inhibitory effect of WEL on TNF-α, NO and PGE_2_ was mediated possibly via the downstream MAPKs pathway but independent of the activation of MAPKs. NF-kB activation rather than the phophorylation of MAPKs may be involved in WEL reduced cytokines production.

## Discussion

WEL belongs to the flavonoids category of phytoestrogens in *Eclipta prostrata* and *Wedelia chinensis.* We investigated its anti-inflammatory activity and underlying mechanism of WEL in LPS stimulated RAW 264.7 cells. WEL has been identified as an anti-inflammatory, growth inhibitory and pro-apoptotic agent in differentiated cells and cancer cells [1,6,9,10]. Previous findings that WEL inhibited IKK activity and caspase-11 expression which resulted in the activation of NF-kB pathway suggested that WEL could be a potential lead compound in anti-inflammatory therapy [9]. However the mechanisms of anti-inflammatory effects of WEL have not been completely delineated. Thus, our study aimed to elucidate the mechanisms underlying the anti-inflammatory effects of WEL.

It is well-known that NO and PGE_2_ play critical roles in the activation of macrophages, and they are closely associated with acute and chronic inflammation [12,26]. Therefore, to study the suppression of NO and PGE_2_ by iNOS and COX-2 is very important in the development of anti-inflammatory agents [23,27]. Here, we demonstrated that WEL can dose-dependently inhibit LPS-induced NO and PGE_2_ production in RAW 264.7 macrophages. Consistent with these findings, WEL also suppressed LPS-induced expression of iNOS and COX-2 at the protein levels in RAW 264.7 macrophages, which suggested that WEL-induced reduction of NO and PGE_2_ may be due to transcriptional suppression of iNOS and COX-2 genes. TNF-α plays a critical role in innate immune responses and it is the principal mediator in responses to LPS stimulated tissue injury and shock. Therefore, we also investigated the effect of WEL on LPS-inducible TNF-α expression [23]. Our results showed that WEL significantly suppressed TNF-α production in LPS-stimulated RAW 264.7 cells.

NF-κB plays a pivotal role in the regulation of the expression of iNOS, COX-2 and inflammatory cytokines such as TNF-α [12]. Activation of NF-κB involves in the phosphorylation and subsequent proteolytic degradation of the inhibitory protein IκB by specific IκB kinases. The free NF-κB (a heterodimer of p50 and p65) then passes into the nucleus, where it binds to NF-κB site in the promoter regions of genes for inflammatory proteins such as cytokines, enzymes, and adhesion molecules [24]. Therefore, we examined the effect of WEL on the phosphorylation of IκB-α and the nuclear translocation of p65 and p50 subunits into the nucleus. Our results showed that LPS treatment caused the decrease of p65 and p50 in the cytoplasm and increase in nucleus, and this effect can be reversed by the pretreatment with WEL in a dose-dependent manner. The present study showed that WEL inhibited LPS-induced NF-κB activation through the suppression of the phosphorylation and degradation of IκB-α and subsequent effects on the nuclear translocation of the subunit of NF-κB in RAW 264.7 macrophages.

The MAPKs pathway is one of the most ancient and evolutionarily conserved signaling pathway and plays essential regulatory roles in both innate and adaptive immune response [17]. MAPKs play an important role in the transcriptional regulation of LPS-induced expression of iNOS and COX-2 [25]. LPS stimulation of cytokines production in human monocytes is involved in several intracellular signaling pathways that include three MAPK pathways: ERK 1 and 2, JNK and p38 and IKK-NF-kB pathway [25]. Several studies have shown that some active compounds inhibit LPS-induced inflammatory cytokines production via the down-regulation of NF-κB and MAPKs activities in RAW 264.7 cells [20,26,28]. Thus, we investigated the effect of WEL on activation of ERK1/2, JNK and p38 in LPS-stimulated cells.

Our results showed that the phophorylation of JNK and ERK in response to LPS were induced with WEL treatment, whereas p38 phosphorylation was not affected. These results indicated that anti-inflammatory mechanism of WEL was mediated possibly via the downstream MAPKs pathway but independent of the activation of MAPK signaling pathway. NF-kB activation rather than the phophorylation of MAPKs may be involved in WEL reduced cytokines production. Similar phenomena were also found in the anti-inflammatory effect of Cucurbitacin E, which was reported by Qiao J [29]. However, the role and the underlying mechanism of WEL-induced activation of MAPKs in LPS-stimulated cells are remained to be further elucidated.

In conclusion, WEL was shown to inhibit the production of NO and PGE_2_ as well as their upstream enzymes iNOS and COX-2 at protein level through inhibition of IκB-α phosphorylation and p65 nuclear translocation in LPS-induced RAW 264.7 cells. The inhibition of iNOS and COX-2 expression was mediated independent of the MAPK.

## Conclusions

The results of our study indicated that WEL exerted anti-inflammatory effects by suppressing the NF-κB pathway. However, the effects of WEL on MAPKs pathway need to be elucidated in further study.

## Abbreviations

WEL: Wedelolactone; LPS: Lipopolysaccharide; DX: Dexamethason; NO: Nitric oxide; PGE2: Prostaglandin E2; L-NAME: N-nitro-L-arginine methyl ester and lipopolysaccharide; iNOS: Inducible nitric oxide synthase; COX-2: Cyclooxygenase-2; TNF-α: Tumor necrosis factor-α; ELISA: Enzyme linked immunosorbent assay; NF-κB: Nuclear factor-kappaB; IκB-α: Inhibitory kappaB alpha; MAPKs: Mitogen-activated protein kinases; ERK: Extracellular signal-regulated kinase; JNK, c-Jun: N-terminal kinase; p-ERK1/2 and p-JNK: Phosphorylation of ERK1/2 and JNK; T-ERK1/2: t-JNK and t-p38, total ERK1/2, JNK and p38.

## Competing interests

The authors declare that they have no competing interests.

## Authors’ contributions

FY and JC made substantial contributions to the conception and design, analysis and interpretation of data, drafting of the manuscript, and were responsible for all experiments. FY and JC contributed equally to this work. Pp S contributed to the cell culture, was involved in molecular analyses of cells (Wester blotting, ELISA, Measurement of TNF–α, NO and PGE_2_). SG performed Transient Transfection and Luciferase Reporter Assay and critically revised the manuscript for important intellectual content. JX contributed to the conception and design, and critically revised the manuscript for important intellectual content. JX contributed to the conception and design, and critically revised the manuscript for important intellectual content. All authors have given final approval of the manuscript.
